# Etiology and Pathogenesis of Marked Elevation of Serum Transaminase in Patients With Acute Gallstone Disease

**DOI:** 10.1155/1991/95059

**Published:** 1991

**Authors:** Masatoshi Isogai, Kitao Hachisuka, Akihiro Yamaguchi, Satoshi Nakano

**Affiliations:** 1 Department of Surgery Ogaki Municipal Hospital, 4-86 Minaminokawa-cho Ogaki 503 Japan; 2 Department of Gastroenterology Ogaki Municipal Hospital, 4-86 Minaminokawa-cho Ogaki 503 Japan

## Abstract

From 1980 through 1988, biliary surgery was performed in 197 patients with acute gallstone disease and concomitant elevation of serum glutamic oxalacetic transaminase (SGOT) or serum glutamic pyruvic transaminase (SGPT) of over 300 Karmen units. In 137 patients, anatomic inspection and liver biopsy were performed during the acute stage of the disease. Impacted and floating bile duct stones were found in 69 (50%) and in 43 (32%) of the 137 patients, respectively. The main liver histology was necrosis of liver cells. After surgery, high serum transaminase fell rapidly with immediate recovery in 99% of the patients. In the remaining 60 patients, their signs and symptoms settled soon after initial conservative treatment and surgery was performed after an average time of 21 days. At laparotomy, impacted bile duct stones were found in 2 (3%) and liver histology revealed regeneration of liver cells.

These findings suggest that marked elevation of serum transaminase in patients with acute gallstone disease might be due to an acute inflammatory liver cell injury caused by impacted bile duct stones or migrating stones, which would be transient and reversible after early resolution of the bile duct obstruction.


*HPB Surgery*, 1991, Vol. 4, pp. 95–107
Reprints available directly from the publisher
Photocopying permitted by license only

© 1991 Harwood Academic Publishers GmbH
Printed in the United Kingdom

### ETIOLOGY AND PATHOGENESIS OF MARKED ELEVATION OF SERUM TRANSAMINASE IN PATIENTS WITH ACUTE GALLSTONE DISEASE

MASATOSHI ISOGAI, KITAO HACHISUKA and AKIHIRO YAMAGUCHI
*Department of Surgery, Ogaki Municipal Hospital, 4–86, Minaminokawa-cho, Ogaki, 503, Japan*

SATOSHI NAKANO
*Department of Gastroenterology, Ogaki Municipal Hospital, 4–86, Minaminokawa-cho, Ogaki, 503, Japan*

*(Received 20 November 1990)*

From 1980 through 1988, biliary surgery was performed in 197 patients with acute gallstone disease and concomitant elevation of serum glutamic oxalacetic transaminase (SGOT) or serum glutamic pyruvic transaminase (SGPT) of over 300 Karmen units. In 137 patients, anatomic inspection and liver biopsy were performed during the acute stage of the disease. Impacted and floating bile duct stones were found in 69 (50%) and in 43 (32%) of the 137 patients, respectively. The main liver histology was necrosis of liver cells. After surgery, high serum transaminase fell rapidly with immediate recovery in 99% of the patients. In the remaining 60 patients, their signs and symptoms settled soon after initial conservative treatment and surgery was performed after an average time of 21 days. At laparotomy, impacted bile duct stones were found in 2 (3%) and liver histology revealed regeneration of liver cells.

These findings suggest that marked elevation of serum transaminase in patients with acute gallstone disease might be due to an acute inflammatory liver cell injury caused by impacted bile duct stones or migrating stones, which would be transient and reversible after early resolution of the bile duct obstruction.

KEY WORDS: Gallstone, pancreatitis, cholangitis, cholecystitis, hepatitis, liver biopsy

### INTRODUCTION

The management of patients with gallstones, especially with bile duct stones, has been facilitated by the measurement of serum alkaline phosphatase or bilirubin. High levels of serum glutamic oxalacetic transaminase (SGOT) and serum glutamic pyruvic transaminase (SGPT), on the other hand, are considered strongly indicative of hepatocellular damage and are usually reliable in the differentiation of hepatocellular from extrahepatic biliary tract diseases. Marked elevation of serum transaminase has been reported in patients with acute gallstone diseases such as acute cholecystitis[1,2], choledocholithiasis[3–6] and gallstone pancreatitis[7,8]. Its mechanism, however, is not yet well understood and there have been controversial interpretations of such phenomena. Without adequate knowledge of the mechanism for such phenomena, high enzyme levels alone might lead to the diagnosis of so called hepatitis and delay in surgical intervention for fear of severe hepatic disease.

Aronsen[9] measured transaminase activity before and after release of choledocal obstruction in dogs and has reported a close association between acute biliary obstruction and high SGOT activity. Association of bile duct obstruction with high SGOT activity has also been reported clinically[3–5,8]. However, the lack of direct observation of the bile duct obstruction by gallstones in a large number of patients prevented wide acceptance of this association.

At our hospital, almost all patients with acute gallstone disease who do not respond to conservative treatment are immediately operated on, provided the patients present a reasonable surgical risk. This provided us with the opportunity to study retrospectively a substantial number of patients with acute gallstone disease and with concomitant marked elevation of serum transaminase during both the acute stage of the disease and the stage of convalescence. The purpose of the study is to report these observations and discuss the etiology and pathogenesis of marked elevation of serum transaminase in patients with acute gallstone disease.

### MATERIAL AND METHODS

Of 2,092 patients operated on for gallstones in Ogaki Municipal Hospital during the last 9 years from 1980 to 1988, 197 patients (9.4%) had severe abdominal pain and concomitant elevation of SGOT or SGPT of over 300 Karmen units and were entered into the study. In all cases, preoperative diagnosis of gallstones was made by ultrasonography or roentogenography using drip infusion cholangiography (DIC), percutaneous transhepatic cholangiography (PTC) or endoscopic retrograde cholangiography (ERC). These patients exhibited no evidence of acute viral hepatitis or other potential causes of transaminase elevation such as heart failure, drug or alcohol abuse.

Patients in the study included: (1) 101 patients who underwent emergency (within 48 hours after admission) and 36 who underwent urgent (over 48 hours) biliary surgery during episodes of severe abdominal pain. They all showed marked elevation of serum transaminase and either showed no objective improvement in response to medical treatment and their general conditions deteriorated or sepsis occurred (137 patients group 1), and (2) 60 patients who received initial conservative management followed by biliary surgery after an average delay of 21 days when their transaminase activities reached near normal levels (group 2). Group 2 patients did not undergo emergency or urgent surgery either because their signs and symptoms settled soon after conservative treatment such as intravenous administration of fluids, antibiotics and analgesics (56 patients), or because impacted bile duct stones were demonstrated by PTC soon after admission, and endoscopic papillotomy (EPT; 1 patient) and percutaneous transhepatic biliary drainage (PTBD; 3 patients) were performed as an emergency procedure.

Fifteen patients (11 group 1 patients and 4 group 2 patients) had concomitant elevation of serum amylase of over 1,000 Caraway units (normal level $<$ 135 C.U.) and the diagnosis of gallstone pancreatitis was made.

There were 71 men and 66 women aged 19 to 87 years old (mean, 54 years) in group 1, while in group 2, 16 were men and 44 were women aged 16 to 83 years old (mean, 52 years). In general, group 1 patients had more severe disease than group 2 patients. Peritonitis and Charcot's triad (abdominal pain, fever and jaundice) were present in 65 (47%) and 24 (18%) of the 137 group 1 patients compared with 11

(18%) and 3 (5%) of the 60 group 2 patients, respectively. The mean level of SGOT (Karmen units, normal < 40 K.U.), SGPT (Karmen units, normal < 35 K.U.), bilirubin (normal < 1.2 mg/dl) and WBC ($\times 10^3$) was 609 ± 433 K.U., 442 ± 232 K.U., 3.8 ± 2.2 mg/dl and 12.9 ± 4.4 in group 1 and 614.9 ± 404 K.U., 545 ± 499 K.U., 3.0 ± 2.1 mg/dl and 8.7 ± 4.2 in group 2, respectively.

In both groups, the findings of surgery, the histological findings of liver biopsy specimens obtained during biliary surgery and the time course of SGOT levels after admission were studied. As a control group, 232 patients during the same study period who had acute gallstone disease but without serum transaminase elevation and underwent emergency biliary surgery were studied and compared with group 1 patients. In the control group, 100 were men and 132 were women aged 21 to 86 years old (mean, 61 years).

Liver biopsy specimens were obtained from 99 patients (68 group 1 patients and 31 group 2 patients) at the time of operation for gallstones. Biopsy specimens were taken from deep within the liver mainly from middle hepatic lobe. This was performed using Silverman's needle immediately after the abdomen was opened. The specimens were fixed immediately, sectioned in the standard manner and stained with hematoxylin and eosin. They were studied under light microscopy. The histological assessment of the specimens was performed blindly by doctors who did not know the clinical course of the patients.

SGOT activities were determined during the first few days after operation in 22 group 1 patients and after relief of pain in 17 group 2 patients. In these patients, the rate of SGOT decrease (apparent half-life of SGOT[10]) was calculated by plotting SGOT activities on semilogarithmic paper.

Results were expressed as Mean ± SD and $\chi^2$ test was used for statistical analysis. Differences were considered significant at $P < 0.05$.

### RESULTS

### *Findings at Operation*

In 69 of the 137 group 1 patients (50%), impacted bile duct stones were found at laparotomy. In 68 patients, no bile duct obstruction was detected at operation, but 43 (32%) had floating bile duct stones. In one of the 25 patients who had stones in the gallbladder only, the intraoperative cholangioscopy demonstrated ampullary injury. This finding was thought to be a residual sign of previous ampullary obstruction by a stone which might have migrated into the duodenum because a gallstone identical to those in the gallbladder was recovered from her feces after operation. In the remaining 24 patients with gallstones in the gallbladder only, postoperative screening of the feces was not performed. There were significant differences in the presence or absence of the bile duct stones between group 1 and control group (Table 1, $P < 0.01$).

In the 69 bile duct obstructions, three different patterns of bile duct obstruction emerged as a result of the investigation of PTC (31 cases), operative findings including operative cholangiography (26 cases) or ERC (12 cases) (Figure 1). All types were due to impaction of one stone in each and presented the typical cholangiographic image of a reversed meniscus in the bile duct. Type A (63 cases) was due to impaction either high above or around the ampulla where the pancreatic

**Table 1** Operative findings – Bile duct stones and acute cholecystitis.

| Group | Cases | *Bile duct stones* △ | | | *Acute inflammation of the gallbladder* △ | | | |
|---|---|---|---|---|---|---|---|---|
| | | *Impacted* | *Floating* | *None** | *Gangrenous* | *Suppurative* | *Serous* | *None* |
| 1 | 137 | 69 (50%) | 43 (32%) | 25 (18%) | 9 (6%) | 20 (15%) | 38 (28%) | 70[2*] (51%) |
| Control | 232 | 0 | 30 (13%) | 202 (87%) | 112 (48%) | 33 (14%) | 42 (18%) | 45 (20%) |

(△: $P<0.01$, group 1 vs control group)
*: Gallstones in the gallbladder only
2*: Including 6 patients who had undergone cholecystectomy previously.

duct obstruction was obscure. Type B (5 cases) was due to ampullary impaction obstructing both the bile duct and the opening of the pancreatic duct. It was confirmed during transduodenal papilloplasty because a gallstone was found lodged at the ampulla of Vater and after the stone was removed, pancreatic juice and bile spouted suddenly. Type C (one case) was due to impaction at the terminal bile duct forming a common channel. Type $A_1$ and Type $B_1$ had multiple bile duct stones above the obstruction (up to 53, mean, 5).

As shown in Table 2, 7 of the 11 group 1 patients (64%) with gallstone pancreatitis had impacted stones at the lower end of the common bile duct (3 Type A, 3 Type $B_2$ and one Type $B_1$). The extent of the gross pancreatic appearance of the 11 patients was almost normal in one, edematous in 7, necrotizing with pancreatic fluid accumulation in the abdomen in one and details were obscure in the remaining 2 patients.

In most group 1 patients, distension of both the gallbladder and the bile duct was found at operation. The gross appearance of the gallbladder was acute cholecystitis in 67 (49%). The intensity of the gallbladder inflammation, however, was slight and obstruction of the cystic duct was not found in most cases. Acute cholecystitis was significantly more infrequent among group 1 patients than control patients (Table 1, $P < 0.01$).

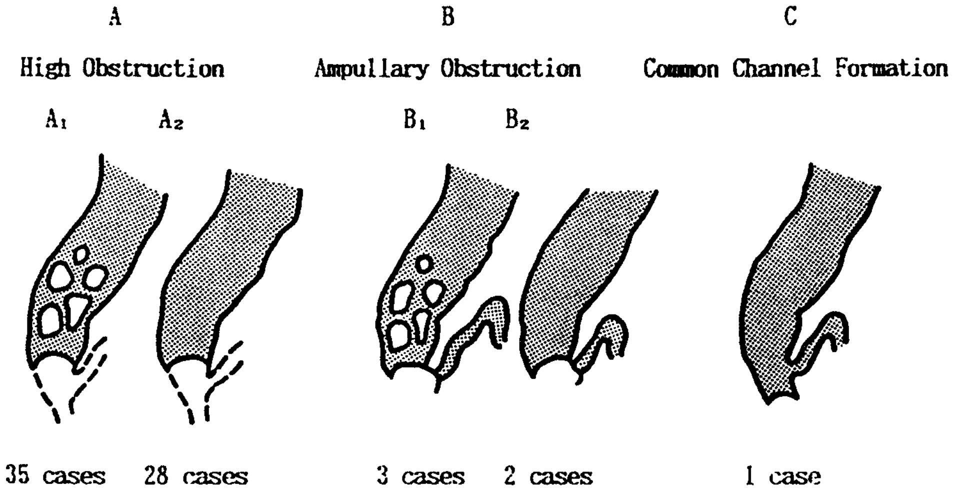

**Figure 1** Types of bile duct obstruction in 69 patients.

**Table 2** Laboratory data on admission and operative findings of 15 patients with serum amylase of over 1,000 Caraway units.

| | | *Laboratory data on admission* | | | *Operative findings* | | |
|---|---|---|---|---|---|---|---|
| *No.* | *Group* | *Amylase** | *SGOT*[2*] | *T.bil*[3*] | *Bile duct stones* | *MPD*[4*] | *Pancreas*[5*] |
| 1 | 1 | 1890 | 995 | 2.7 | Impacted (Type $B_2$) | Obstructed | E |
| 2 | 1 | 1295 | 1305 | 3.2 | Impacted (Type $B_1$) | Obstructed | E |
| 3 | 1 | 1850 | 473 | 2.2 | Impacted (Type $B_2$) | Obstructed | N |
| 4 | 1 | 2090 | 429 | 5.1 | Impacted (Type $B_2$) | Obstructed | E |
| 5 | 1 | 2690 | 429 | 5.1 | Impacted (Type A) | Undetectable | ? |
| 6 | 1 | 2095 | 318 | 4.5 | Impacted (Type A) | Undetectable | E |
| 7 | 1 | 1285 | 609 | 2.3 | Impacted (Type A) | Undetectable | ? |
| 8 | 1 | 2030 | 787 | 1.4 | None[6*] | — | E |
| 9 | 1 | 1380 | 920 | 2.6 | None | — | Nec |
| 10 | 1 | 1518 | 375 | 3.6 | None | — | E |
| 11 | 1 | 3170 | 692 | 8.7 | None | — | E |
| 12 | 2 | 1120 | 970 | 3.0 | None | — | E |
| 13 | 2 | 1915 | 1115 | 4.0 | None | — | N |
| 14 | 2 | 2615 | 1386 | — | None | — | E |
| 15 | 2 | 1680 | 1346 | — | None | — | N |

*: Caraway units (N<135 C.U.)
[2*]: Serum glutamic oxalacetic transaminase (N<40 K.U.)
[3*]: Total bilirubin (N<1.2 mg/dl)
[4*]: Main pancreatic duct
[5*]: N; normal, E; edematous, Nec; necrotizing, ?; obscure
[6*]: Gallstones in the gallbladder alone

Operative findings of group 2 patients, on the other hand, were as follows: (1) impacted bile duct stones were found in 2 (3%) who had choledochoduodenal fistula, floating bile duct stones in 21 (35%) and gallstones in the gallbladder only in 37 (62%), (2) acute cholecystitis was present in only 4 (7%), (3) gross pancreatic appearance showed only slight changes, appearing almost normal or edematous in 4 who had gallstone pancreatitis.

Bacteriological examination of the bile obtained during operation was performed for 107 patients. Bile culture was positive in 61 (72%) of the 85 group 1 patients and in 10 (45%) of the 22 group 2 patients. Escherichia coli was the most frequent organism followed by Klebsiella pneumonia and Streptococcus faecalis.

### *Histological Findings of the Liver Biopsy Specimens*

The main histological changes of the liver biopsy specimens of group 1 were degeneration and necrosis of liver cells and acute cholangitis. Necrosis of liver cells was defined as an accumulation of neutrophils in an area where liver cells had vanished from liver cell plates (hepatocytolysis and cellular infiltration[11]). Acute cholangitis was defined as neutrophil infiltration around and into the lumen of the bile duct in the portal triad[12]. These findings were graded as: (-) none or negligible, (±) slight, (+) moderate and (++) marked. Marked or moderate degree of liver cell necrosis and acute cholangitis were observed in 55 (81%) and in 57 (84%), respectively (Table 3). Degeneration and necrosis of liver cells extended intralobularly and were not confined to the periportal area. Cholestasis was commonly seen and marked dilatation of the bile canaliculus bounding the injured liver cells was often detected at high magnification (Figure 2). There was no correlation between the level of serum transaminase and the severity of liver histological findings.

**Table 3** Findings of liver biopsy specimens

| | | *Necrosis of liver cells** | | | | *Acute cholangitis** | | | | |
|---|---|---|---|---|---|---|---|---|---|---|
| *Group* | *Cases* | *(++)* | *(+)* | *(±)* | *(−)* | *(++)* | *(+)* | *(±)* | *(−)* | *(?[2*])* |
| 1 | 68 | 22(32%) | 33(49%) | 13(19%) | 0 | 19(28%) | 38(56%) | 5(7%) | 1(2%) | 5(7%) |
| 2 | 31 | 1[3*](3%) | 12(39%) | 12(39%) | 6(19%) | 0 | 2(7%) | 8(26%) | 15(48%) | 6(19%) |

*: (++), Marked; (+), Moderate; (±), Slight; (−), None or negligible
[2*]: Portal tract was not included in biopsy specimen.
[3*]: A patient with an impacted bile duct stone and cholecystoduodenal fistula.

**Figure 2** Marked dilatation of the bile canaliculus. Marked dilatation of the bile canaliculus bounding the injured liver cells was often detected in the liver biopsy specimens of group 1 patients (H & E × 1,000). (*See colour plate at the back of this publication*).

In group 2, on the other hand, necrosis of liver cells and acute cholangitis were less common (Table 3) and dense and cobblestone-like liver cells indicating liver cell regeneration[13] were often seen.

*Time Course of SGOT*

After biliary surgery, a rapid fall in serum transaminase activities and an immediate remission of symptoms were observed in almost all group 1 patients. In group 2, serum transaminase activities dropped and symptoms were relieved soon after admission either by conservative treatment (56 patients) or after relief of bile duct obstruction by EPT (one patient) or PTBD (3 patients). In both groups, SGOT activities returned to near normal levels within one week in most patients. The level of SGOT was 43.5 ± 22.6 K.U. at the 7th postoperative day for group 1 and 39.7 ± 25.1 K.U. at the 7th day in hospital for group 2 (Figure 3). In 39 patients in

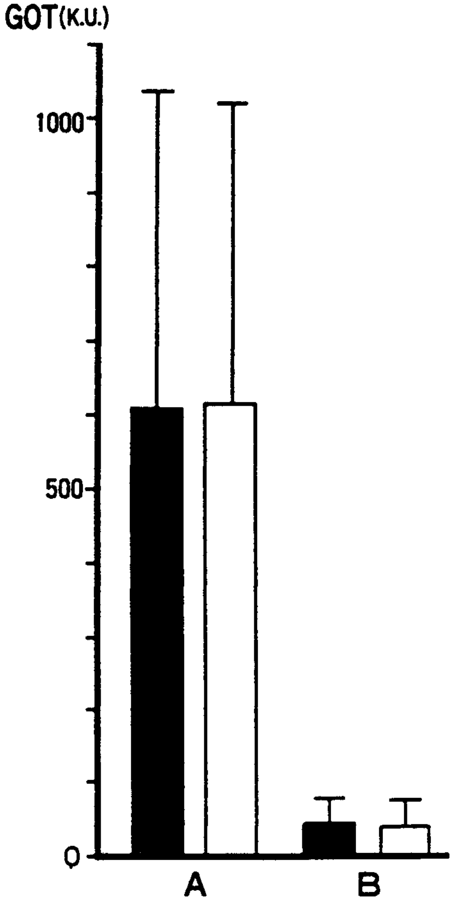

**Figure 3** SGOT levels in patients of group 1 (■) and group 2(□) Levels on the day of admission (A), and on the 7th postoperative day for group 1 and on the 7th day in hospital for group 2 (B). Bars indicate 1 SD.

whom SGOT activities were determined during the first few days after operation (22 group 1 patients) or after relief of pain (17 group 2 patients), SGOT activities decreased in a straight line on semilogarithmic paper; the apparent half-life of SGOT was 0.66 ± 0.21 days in group 1 and 0.73 ± 0.17 day in group 2, respectively.

### *Operative Procedures and Results of Surgical Treatment*

Operative procedures and the results of surgical treatment of both groups are given in Table 4. Biliary surgery consisted of cholecystectomy and operative cholangiography, with choledocholithotomy followed by T-tube decompression of the common bile duct or drainage procedures such as papilloplasty or choledochoduodenostomy if common bile duct stones were present. In group 1 patients, transduodenal papilloplasty was mainly performed when it was difficult to remove impacted ampullary stones.

**Table 4** Operative procedures and results of surgical treatment.

| | *Group 1 (137 cases)* | *Group 2 (60 cases)* |
|---|---|---|
| Operative procedures | | |
| (1): Cholecystectomy | 22 | 34 |
| (2): (1) + Choledocholithotomy | 51(1) | 13 |
| (3): (2) + Papilloplasty | 58(1) | 10(1) |
| (4): (2) + Choledochoduodenostomy | 6(4) | 3 |
| Mortality | 1.5% | 0% |
| Hospital stay (days*) | 34.4 ± 8.5 | 39.6 ± 12.3 |

( ): Number of patients who had undergone cholecystectomy previously.
*: Mean ± SD

There were 2 deaths only in group 1: an 80-year-old male with Reynold's pentad (Charcot's triad plus hypotension and depressed sensorium) underwent cholecystectomy and choledocholithotomy with T-tube decompression of the bile duct but died of irreversible shock due to acute obstructive suppurative cholangitis one day postoperatively. Another patient was a 77-year-old female with necrotizing pancreatitis and accumulation of pancreatic fluid in the abdomen. After cholecystectomy with drainage of her abdominal cavity, she developed a retroperitoneal abscess and died of multi-organ failure (MOF) 45 days postoperatively. In the majority of patients, however, the postoperative course was uneventful. Hospital stay days were 34.4 ± 8.5 in group 1 and 39.6 ± 12.3 in group 2, respectively.

### DISCUSSION

The presence of impacted bile duct stones during episodes of severe abdominal pain with marked elevation of serum transaminase in 69 (50%) of the 137 group 1 patients who underwent emergency or urgent biliary surgery supports a close association of acute biliary obstruction and high SGOT activity[3-5,8,9]. The absence of impacted bile duct stones in 43 patients (32%) with floating bile duct stones was probably due to passage of the initial impacted stones into the duodenum which might have caused transaminase elevation. Of greater concern, however, were the 25 attacks (18%) in which serum transaminase activities were elevated markedly but without evidence of bile duct stones on subsequent investigation. The uncertainty of the reliability of negative biliary tract radiology has been known and Mayer *et al.*[8] have reported that they have retrieved stones from 11 patients who had negative radiologic investigations. Acosta *et al*[14] and Kelly[15] have demonstrated that gallstones were recovered from the stool in 31% of 29 patients and 86% of 134 patients with gallstone pancreatitis who were suspected to have migrating stones. In one patient in our series who had stones in the gallbladder only at operation, a gallstone identical to those found in the gallbladder was recovered from her stool after operation. This suggests that the 25 attacks without evidence of bile duct stones might be due to missing or migrating stones.

Though Iwasaki *et al.*[10] have reported that when SGOT activities decreased by half in about 0.6 day, the pathological factors attributed to transaminase elevation were thought to have disappeared, rapid falls in SGOT of 50% in about 0.6 day occurred after emergency or urgent biliary surgery in our series, so the factors responsible for SGOT elevation were thought to have disappeared after biliary surgery. This analysis of SGOT disappearance rate also supports a close association between acute biliary obstruction and high SGOT activity.

Serum transaminase elevation has been reported in patients with acute cholecystitis[1,2]. In our series, gross appearance of the gallbladder showed acute cholecystitis in 67 (49%) of the 137 group 1 patients. In the majority of patients, however, the intensity of the gallbladder inflammation was slight and obstruction of the cystic duct was not found. Acute cholecystitis was significantly more infrequent among group 1 patients than control patients ($P < 0.01$). Accordingly, acute inflammation of the gallbladder was thought to be secondary to bile duct obstruction and not to be the initial process responsible for transaminase elevation.

The findings of the liver biopsy specimens taken during the acute stage of the disease (group 1) were hepatocellular necrosis and degeneration of liver cells. It has been reported that increased plasma transaminase activity is a sensitive indicator of liver cell damage, and early elevation is usual in diseases that produce hepatocellular injury[16]. Therefore, marked elevation of serum transaminase activity in patients with acute gallstone disease is probably due to liver cell injury which might be caused by acute bile duct obstruction.

It has been reported that liver cell necrosis does not release large amounts of alkaline phosphatase into the circulation, and that the high levels associated with cholestasis are probably due to a combination of regurgitation via the sinusoids and increased synthesis in the bile canaliculi[17]. Thus alkaline phosphatase rises in parallel with bilirubin, and a significant elevation may not occur if the bile duct is obstructed only transiently. A marked elevation of alkaline phosphatase and bilirubin in patients with gallstones may be a sign of prolonged bile duct obstruction[8]. Though plasma samples for alkaline phosphatase were not taken in the majority of patients in the study, serum bilirubin level was $3.8 \pm 2.2$ mg/dl in group 1 and $3.0 \pm 2.1$ mg/dl in group 2 and was not markedly elevated.

The morphological change of the liver in acute bile duct obstruction has not been reported in detail and a rise in serum transaminase activity has been supposed unrelated to hepatic necrosis[3–5]. Mossberg and Ross[3] have suggested that bile duct obstruction could provoke augmented hepatic production and/or release of transaminase with leakage of the enzyme from grossly intact hepatocytes. Shora and

Donovitch[4] have also suggested that transaminase activity could diffuse out of leaky cell membranes which might result from bile duct obstruction. In these papers, however, little histological findings at the time of maximal SGOT activity have been reported because liver biopsy specimens were obtained after a long interval between maximal SGOT activity and operation.

The rapid falls in SGOT after admission in group 2 patients might indicate that there was a temporary obstruction of the bile duct by a passing stone. Accordingly, the liver biopsy specimens of group 2 patients which were taken after an average time of 21 days from admission can be considered to be the liver histology during the stage of convalescence of the disease. The histological features of this disease were degeneration and necrosis of liver cells in the acute stage (group 1) and regeneration of liver cells in the stage of convalescence (group 2), which are thought to be an acute inflammatory reaction of the liver to the injury caused by impacted bile duct stones.

The mechanism of liver injury caused by acute bile duct obstruction remains unclear. Marked dilatation of the bile canaliculus bounding the injured liver cells (Figure 2) led us to speculate that if the bile duct is obstructed by impacted stones, it becomes a closed system filled with bile and that pathological changes in the bile duct such as bile stasis, increased pressure or infection may affect the liver cells which bound the bile canaliculus and cause hepatocellular injury. Mayer *et al.*[8] have reported that transient ampullary obstruction causes a rapid rise in bile duct pressure and consequent liver cell damage. A combination of bile stasis and inflammation also has been reported to cause a mechanical insufficiency of lymph circulation, leading to extensive liver cell necrosis[18].

Saharia and Cameron[19] have reported that almost all patients with acute cholangitis had elevation of serum transaminase and this was a reflection of the pathological condition of the bile duct. In the majority of patients of their series, however, the SGOT levels were between 20 and 100 units (I.U./L) and were not markedly elevated. Charcot's triad was present in 14% on admission and histological findings of acute cholangitis was detected in 91% during the acute stage in our series. However, cholangitis is thought to be subsequent to bile duct obstruction and not to be the initial process responsible for marked transaminase elevation.

In 11 groups 1 patients with gallstone pancreatitis, 7 (64%) had impacted stones at the lower end of the common bile duct and in 4 of the 7, it was confirmed that gallstones had lodged at the ampulla obstructing both the bile duct and the pancreatic duct. These observations are consistent with the concept that gallstone pancreatitis is caused by the migration of a stone into or through the ampulla of Vater[20].

In summary, marked elevation of serum transaminase in patients with acute gallstone disease might be due to an acute inflammatory liver cell injury caused by impacted bile duct stones or migrating stones (gallstone hepatic injury or gallstone hepatitis[21]), which would be transient and reversible after early resolution of the bile duct obstruction. Marked elevation of serum transaminase on the day of admission in patients with gallstones has turned our attention from hepatocellular to extrahepatic biliary tract diseases such as impacted bile duct stones including passing or missing stones, acute cholecystitis or acute cholangitis secondary to bile duct obstruction and gallstone pancreatitis.

*Acknowledgements*

The authors are grateful to Shigehiko Shionoya, M.D., Yuji Nimura, M.D., and Akihiro Yasui, M.D. for helpful criticisms.

### *References*

1. Adames, J.T., Clermont, G.H., Schwartz, S.I. and Rochester, N.Y. (1970) Acute cholecystitis and serum transaminase activity. *Arch. Surg.* **101**, 366–369
2. Adames, J.T., Clermont, G.H. and Schwartz, S.I. (1970) Serum glutamic oxalacetic transaminase activity in cholecystitis. *Surgery*, **68**, 492–497
3. Mossberg, S.M. and Ross, G. (1963) High serum transaminase activity associated with extrahepatic biliary disease. A clinical and pathological study of sixty patients with serum glutamic oxalacetic transaminase levels of 300 units or greater. *Gastroenterol*, **45**, 345–353
4. Shora, W. and Donovitch, S.H. (1969) Marked elevation of serum transaminase activities in extrahepatic biliary tract disease. *Am. J. Gastroenterol*, **61**, 575–585
5. Ginsberg, A.L. (1970) Very high levels of SGOT and LDH in patients with extrahepatic biliary tract obstruction. *Dig. Dis.*, **15**, 803–807
6. Anciaux, M.L., Pelletier, G., Attali, P., Liguory, C. and Etienne, J.P. (1986) Prospective study of clinical and biochemical features of symptomatic choledocholithiasis. *Dig. Dis. Sci.*, **31**, 449–453
7. McMahon, M.J. and Pickford, I.R. (1979) Biochemical prediction of gallstones in an attack of acute pancreatitis. *Lancet*, **15**, 541–543
8. Mayer, A.D. and McMahon, M.J. (1985) Biochemical identification of patients with gallstones associated with acute pancreatitis on the day of admission to hospital. *Ann. Surg.*, **201**, 68–75
9. Aronsen, K.F. (1961) Liver function studies during and after complete extra-hepatic biliary obstruction in dogs. *Acta Chir. Scand. Suppl.* **275**, 1–113
10. Iwasaki, Y., Ohkubo, A. and Kamei, S. (1978) Serial determination of serum GOT isozyme activities for the evaluation of acute hepatic damage. *Jpn. J. Gastroenterol*, **75**, 34–43
11. Edomondson, H.A., Schift, L. and Schift, E.R. (1982) Needle Biopsy of the Liver, *Diseases of the Liver*, 5th ed. Philadelphia: Lippincott
12. O'Connor, M.J. and Sumner, H.W. (1982) The clinical and pathological correlation in mechanical biliary obstruction and acute cholangitis. *Ann. Surg.*, **195**, 419–423
13. Uchida, T. (1983) Acute Viral Hepatitis, *Viral Hepatitis* — Histological Features and Differential Diagnosis-, Tokyo: Chugai Igaku Co.
14. Acosta, J.M., Pellegrini, C.A. and Skiner, D.B. (1980) Etiology and pathogenesis of acute biliary pancreatitis. *Surgery*, **88**, 118–125
15. Kelly, T.R. (1980) Gallstone pancreatitis: The timing of surgery. *Surgery*, **88**, 345–350
16. Clermont, R.J. and Chalmers, T.C. (1967) The transaminase tests in liver disease. *Medicine*, **46**, 197–207
17. Kaplan, M.M. (1972) Alkaline phosphatase. *N. Engl. J. Med.*, **286**, 200–202
18. Rusznyák, I., Földi, M. and Szabó, G. (1967) The liver, Lymphatics and Lymph Circulation, Physiology and Pathology, London: Pergamon Press
19. Saharia, P.C. and Cameron, J.L. (1976) Clinical management of acute cholangitis. *Surg. Gynecol. Obstet.* **142**, 369–372
20. Howard, J.M. (1987) Gallstone Pancreatitis, Surgical Diseases of the Pancreas. Philadelphia: Lea & Febiger
21. Isogai, M. (1985) A clinicopathological study on the pathogenesis of hepatitis caused by gallstone. *Jpn. J. Gastroenterol. Surg.*, **18**, 1650–1658

*(Accepted by S. Bengmark on 20 November 1990)*

### INVITED COMMENTARY

The differentiation between extrahepatic bile duct obstruction and a hepatocellular process in patients with hyperbilirubinemia is important. Raised levels of serum transaminases, AST(SGOT) and ALT(SGPT) usually indicate hepatocellular damage while raised levels of alkaline phosphatase support a diagnosis of biliary tract obstruction or malignancy. These biochemical tests are useful in conjunction with the history of disease, signs and symptoms. Serum transaminases, however, may be raised in several conditions not related to hepatocellular damage and also in acute cholecystitis (1) and extrahepatic biliary obstruction (2–5). The time course of these biochemical abnormalities are important and changes in serum transaminases are known to be faster than those of serum bilirubin and alkaline phosphatases. The presence of high serum transaminase levels in the early course of gallstone-induced common bile duct obstruction has recently been discussed (5) and enzyme leakage into the blood from hepatocytes due to reduced transport or transient hepatocellular necrosis was suggested as an underlaying mechanism.

The present study is an attempt to clarify the cause of a marked elevation of serum transaminase in patients with acute gallstone disease. The authors divide a large collection of patients with acute gallstone-related disease in two groups, one with patients treated with emergency surgery and one with patients treated with conservative management followed by surgery after an average time of 21 days. Serum transaminases, clinical findings and liver histology were studied in these two groups. At admission the SGOT levels were strongly raised, averaging 600 (Karmen units), in both groups. In the acutely operated group the histological picture demonstrated degeneration and necrosis of liver cells and cholangitis but there was no correlation between the level of serum transaminase activity and the severity of the histologic abnormalities. The raised SGOT levels declined close to normal values in a week. In patients exposed to a late operation histologic abnormalities were less common and morphologic signs of regeneration were seen. The authors draw the conclusion that marked elevation of serum transaminase is often seen in patients with acute gallstone disease and might be due to a liver cell injury caused by impacted or migrating gallstones in the common bile duct.

The message of this study that serum transaminases may be raised in the very early stage of biliary obstruction is clinically important. Further, it is an interesting observation that biliary obstruction caused by gallstones may cause morphologic signs of hepatocyte damage. The design of the study, however, makes it difficult to draw firm conclusions about the underlaying mechanism causing high levels of serum transaminases in the early course of acute gallstone disease. Half of the patients operated acutely had acute cholecystitis but this was seen only in a few in the group operated after three weeks. The presence of an acutely inflamed gallbladder may well have caused liver cell damage and could thus also be responsible for the raised levels of SGOT. To differentiate between these possibilities further, more clearly designed studies are needed.

### *References*

1. Mossberg, S.M. and Ross, G. (1963) High serum transaminase activity associated with extrahepatic biliary disease. A clinical and pathological study of sixty patients with serum glutemic oxalacetic transaminase levels of 300 units or greater. *Gastroenterology*, **45**, 345–353

2. Ginsberg, A.L. (1970) Very high levels of SGOT and LDH in patients with extrahepatic biliary tract obstruction. *Dig. Dis.* **15**, 803–807
3. Fortson, W.C., Tedesco, F.J., Starnes, E.C. *et al.* (1985) Marked elevation of serum transaminase activity associated with extrahepatic biliary tract disease. *J. Clin. Gastroenterol*, **7**, 502–505
4. Anciaux, M.L., Pelletier, G., Attali, P. *et al.* (1986) Prospective study of clinical and biochemical features of symptomatic choledocholithiasis. *Dig. Dis. Sci.* **31**, 449–453
5. Patwardhan, R.V., Smith, O. and Farmelant, M.H. (1987) Serum transaminase levels and cholescintigraphic abnormalities in acute biliary tract obstruction. *Arch. Int. Med.* **147**, 1249–1253

Joar Svanvik
Dept. of Surgery
Sahlgrens' Hospital
Gothenburg, Sweden

